# Mixed Convection Flow of Nanofluid in Presence of an Inclined Magnetic Field

**DOI:** 10.1371/journal.pone.0073248

**Published:** 2013-09-23

**Authors:** Saima Noreen, Bashir Ahmed, Tasawar Hayat

**Affiliations:** 1 Department of Mathematics, Comsats Institute of Information Technology, Islamabad, Pakistan; 2 Department of Mathematics, Faculty of Science, King Abdulaziz University, Jeddah, Saudi Arabia; 3 Department of Mathematics, Quaid-i-Azam University, Islamabad, Pakistan; University Paul Sabatier, France

## Abstract

This research is concerned with the mixed convection peristaltic flow of nanofluid in an inclined asymmetric channel. The fluid is conducting in the presence of inclined magnetic field. The governing equations are modelled. Mathematical formulation is completed through long wavelength and low Reynolds number approach. Numerical solution to the nonlinear analysis is made by shooting technique. Attention is mainly focused to the effects of Brownian motion and thermophoretic diffusion of nanoparticle. Results for velocity, temperature, concentration, pumping and trapping are obtained and analyzed in detail.

## Introduction

Peristaltic motion is now an important research topic due to its immense applications in engineering and physiology. This type of rhythmic contraction is the basis of peristaltic pumps that move fluids through tubes without direct contact with pump components. This is a particular advantage in biological/medical applications where the pumped material need not to contact any surface except the interior of the tube. The word “peristalsis” comes from a Greek word “Peristaltikos”which means clasping and compressing. The peristaltic flow has specific involvement in the transport of urine from kidney to the bladder, chyme movement in gastrointestinal tract, movement of ovum in the female fallopian tubes, blood circulation in the small blood vessels, roller and finger pumps, sanitary fluid transport and many others. Latham [1] and Shapiro et al. [2] reported initial studies for the peristaltic flow of viscous fluid. Since then ample attempts have been made for peristalsis in symmetric flow configuration (see few recent studies [3]–[8]). On the other hand the physiologists argued that the intra-uterine fluid flow (because of mymometrical contractions) represents peristaltic mechanism and the myometrical contractions may appear in both asymmetric and symmetric channels [9]. Hence some researchers [10]–[15] discussed the peristaltic transport in an asymmetric channel with regard to an application of intra-uterine fluid flow in a nonpregnant uterus.

Heat transfer in cooling processes is quite popular area of research in industry and medical science. Conventional methods for increasing cooling rates include the extended surfaces such as fins and enhancing flow rates. These conventional methods have their own limitations such as undesirable increase in the thermal management system's size and increasing pumping power respectively. The thermal conductivity characteristics of ordinary heat transfer fluids like oil, water and ethylene glycol mixture are not adequate to meet today's requirements. The thermal conductivity of these fluids have key role in heat transfer coefficient between the heat transfer medium and heat transfer surface. Hence many techniques have been proposed for improvement in thermal conductivity of ordinary fluids by suspending nano particles in liquids. The term “nano” introduced by Choi [16] describes a liquid suspension containing ultra-fine particles (diameter less than 50 nm). The nanoparticle can be made of metal, metal oxides, carbide, nitride and even immiscible nano scale liquid droplets. Natural convective boundary-layer flow in a porous medium saturated by a nanofluid is studied by Nield and Kuznetsov [17]. Although the literature on flow of viscous nanofluid has grown during the last few years but the information regarding peristaltic flow of nanofluid is almost nonexistant. For example, Akbar et al. [18] studied the influence of partial slip in peristaltic flow of viscous fluid.

The aim of present study is to venture further in the regime of peristalsis for fluids with nanoparticles. Therefore we examine here the mixed convective peristaltic transport of nanofluid in an inclined asymmetric channel in the presence of inclined magnetic field. Channel asymmetry is produced by peristaltic waves of different amplitude and phases. Mathematical modelling involves the consideration of Brownian motion and thermophorsis effects. Numerical solution of nonlinear problem is obtained using shooting method. Limiting case for nanofluid in symmetric channel is also analyzed. Detailed analysis for the quantities of interest is seen.

## Mathematical Formulation

We consider mixed convective viscous nanofluid in an inclined asymmetric channel of width 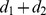. The fluid is conducting in presence of inclined magnetic field B_0_ only. Let 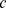 be the speed by which sinusoidal waves propagate along the channel walls. The 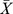 and 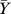-axes in the rectangular coordinates 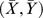 system are taken parallel and transverse to the direction of wave propagation. Further the lower wall has temperature 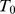 and nanoparticle concentration 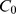 while the temperature and nanoparticle concentration at the upper wall are denoted by 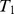 and 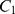 respectively. The geometry of wall surfaces can be represented as follows:
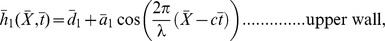


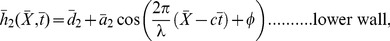
(1)where 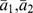 are the wave amplitudes and the phase difference 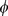 varies in the range 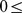 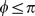. The case 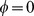 is subject to the symmetric channel with waves out of phase and the waves are in phase when 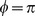. Here 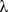 is the wavelength, 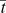 the time and 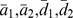 and 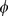 satisfy 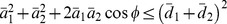. Denoting the velocity components 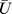 and 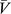 along the 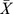 and 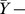 directions in the fixed frame, we can represent velocity 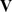 in following definition:
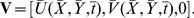
(2)


The equations governing the flow under consideration are
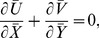
(3)

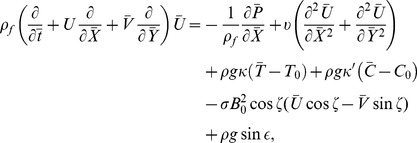
(4)

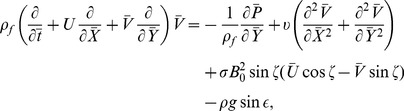
(5)

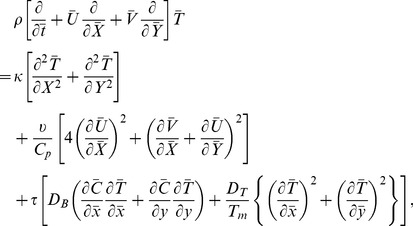
(6)

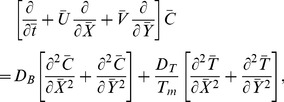
(7)in which 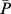 is the pressure, 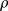 the density of fluid, 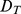 the thermophoretic diffusion coefficient, *g* the acceleration due to gravity, *T* the temperature, 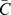 the concentration, 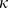 the thermal conductivity, 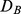 the Brownian diffusion coefficient, 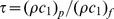 the ratio of the specific heat capacity of the nanoparticle material and heat capacity of the fluid, 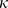 the thermal diffusivity, 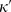 the thermal diffusivity, 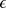 the angle of channel inclination, 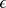 the channel inclination, 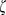 the inclined magnetic field, 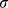 the electrical conductivity, 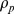 is the density of the particle, and 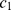 is the volumetric volume expansion coefficient

The transformations between fixed and wave frames are defined as follows:
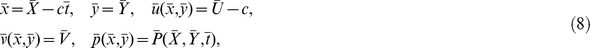
(8)in which (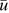) and 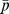 are the velocity components and pressure in the wave frame.

We now introduce
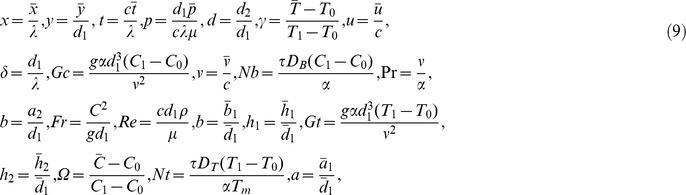
(9)where 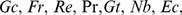 represent the local mass Grashof number, Frude number, Reynolds number, Prandtl number, local temperature Grashof number, Brownian motion parameter, Eckert number and thermophoresis parameter respectively.

Employing transformation (8), dimensionless variables (9) and long wavelength and low Reynolds number 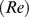 approximation, the dimensionless forms of above equations in terms of stream function 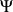


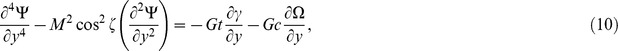
(10)

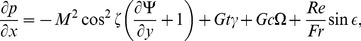
(11)

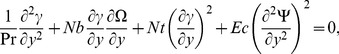
(12)

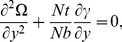
(13)where
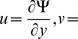
The dimensionless boundary conditions are given by
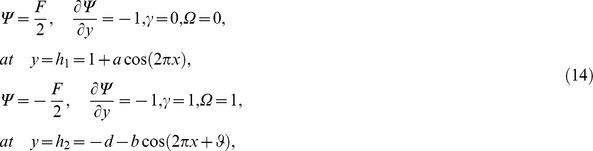
(14)with 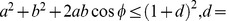. The dimensionless time mean flow rate *F* in the wave frame is related to the dimensionless time mean flow rate 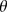 in the laboratory frame by the following expressions
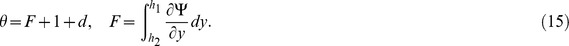
(15)


## Results and Discussion

Our main interest in this section is to examine the velocity (*u*), temperature (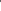), concentration (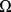) and pressure rise per wavelength (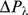) for the influence of local Grashof number (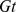), Frude number (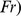, mass Grashof number (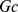), Prandtl number (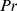), Eckert number (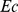), Brownian motion parameter (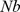), Hartman number (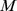), phase difference parameter (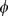) and thermophoresis parameter (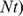.

### 3.1 Pumping characteristics

**Figure 1:**
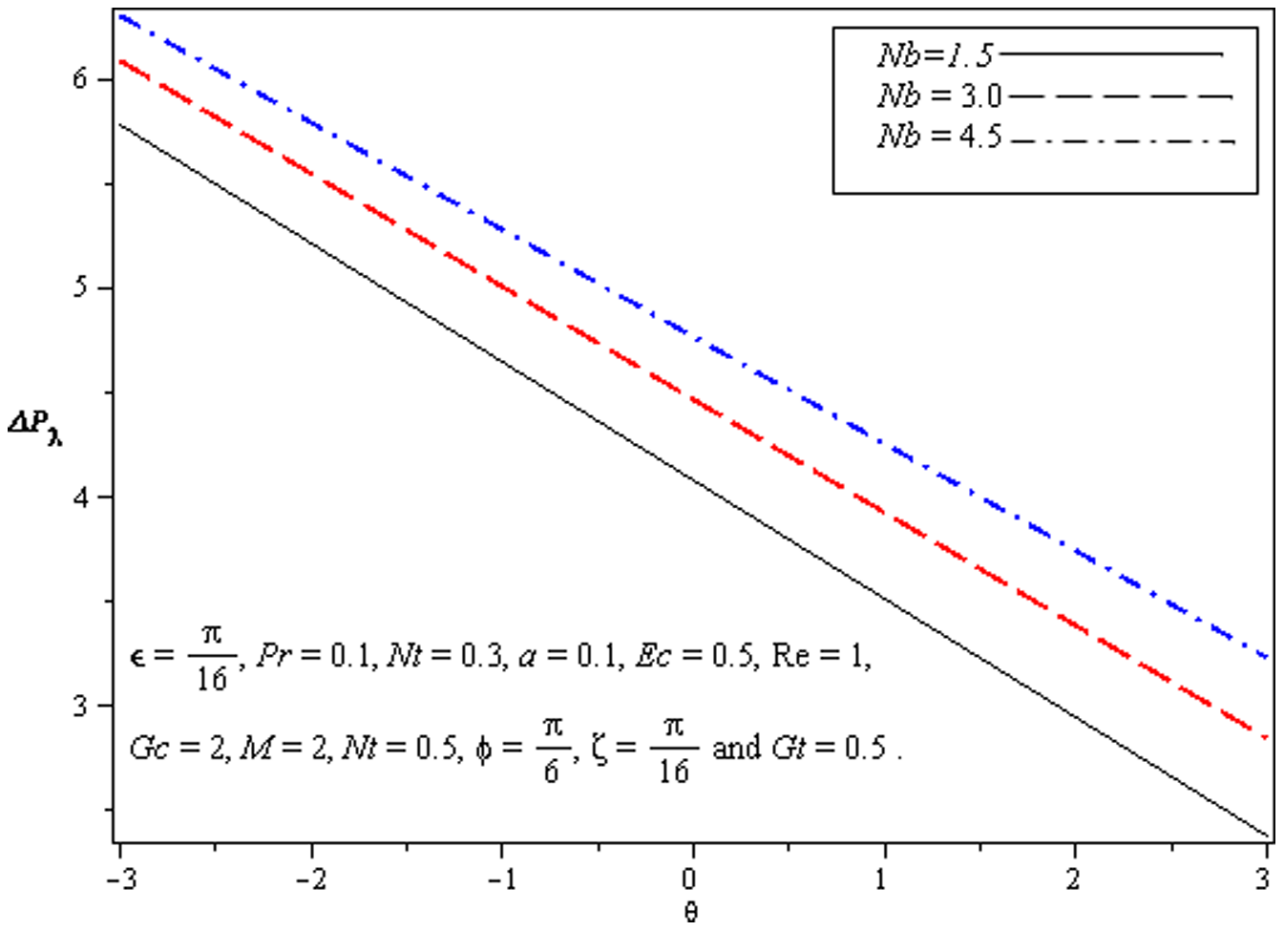
Influence of *Nb* on Pressure rise 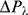.

**Figure 2:**
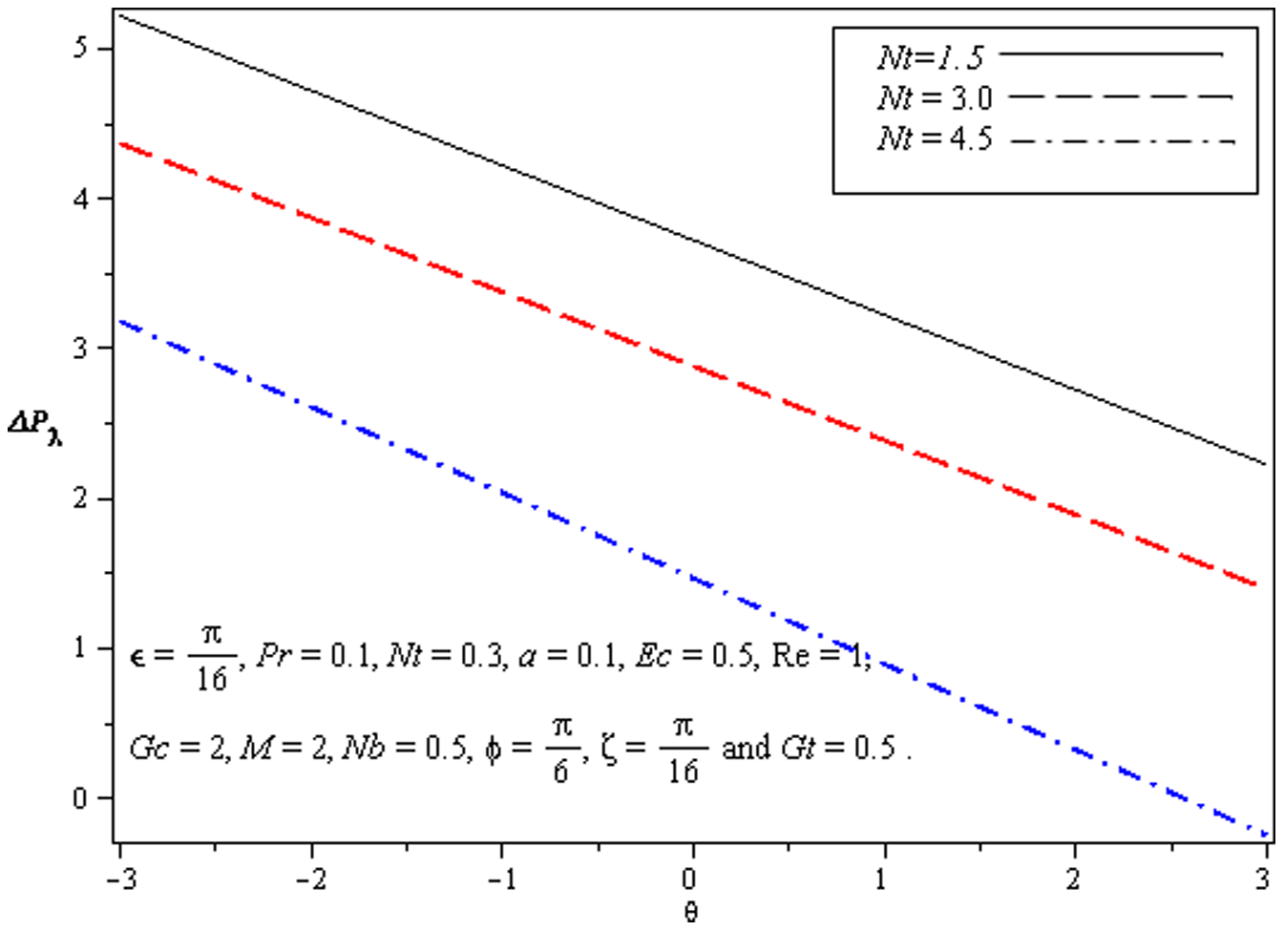
Influence of *Nt* on Pressure rise 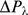.

**Figure 3:**
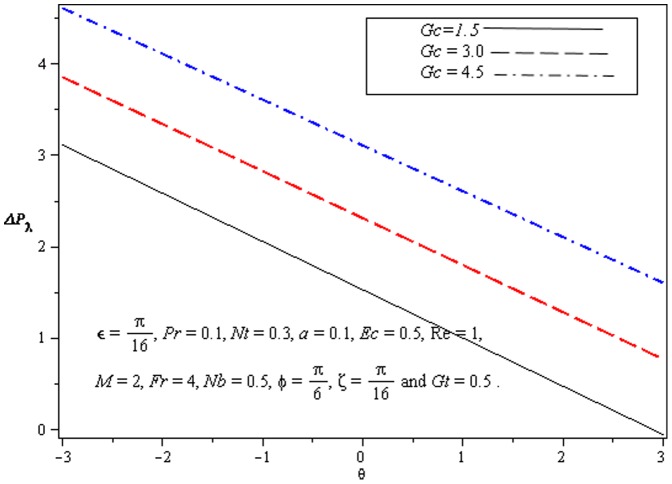
Influence of *Gc* on Pressure rise 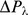.

This subsection illustrates the behavior of emerging parameters 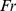, 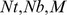, and 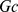 on pressure rise per wavelength 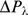. The dimensionless pressure rise per wavelength versus time-averaged flux 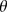 has been plotted in the Figs. 1–3. Here the upper right-hand quadrant 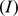 denotes the region of peristalsis pumping, where 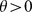 (positive pumping) and 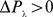 (adverse pressure gradient). Quadrant 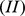, where 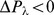 (favorable pressure gradient) and 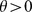 (positive pumping), is designated as augmented flow (copumping region). Quadrant 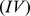, such that 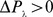 (adverse pressure gradient) and 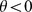, is called retrograde or backward pumping. The flow is opposite to the direction of the peristaltic motion and there is no flow in the last (Quadrant 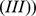. There is an inverse linear relation between 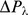 and 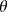. Pumping rate decreases by increasing 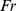 in pumping region. Figs. 1 and 2 show that 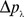 decreases with 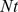 and increases with 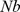 in all the pumping regions. This is due to the reason that Brownian diffusion is directly related to increased flow rate. It is noticed from Fig. 3 that 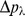 increases with 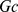 in all the pumping regions by fixing the values of other parameters i.e Mass convection supports pressure rise in pumping region.

### 3.2 Flow characteristics

**Figure 4:**
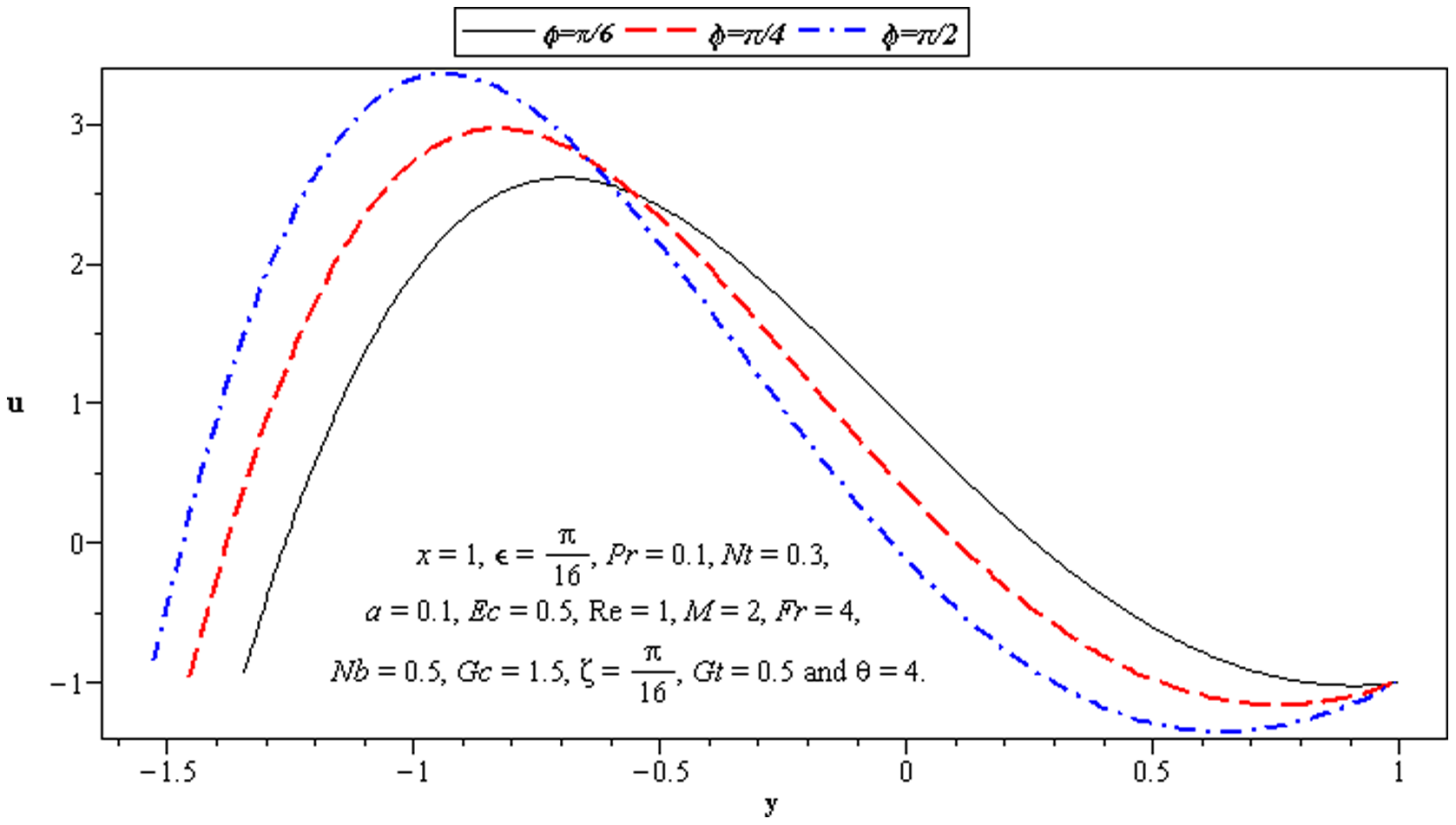
Influence of *φ* on velocity *u*.

**Figure 5:**
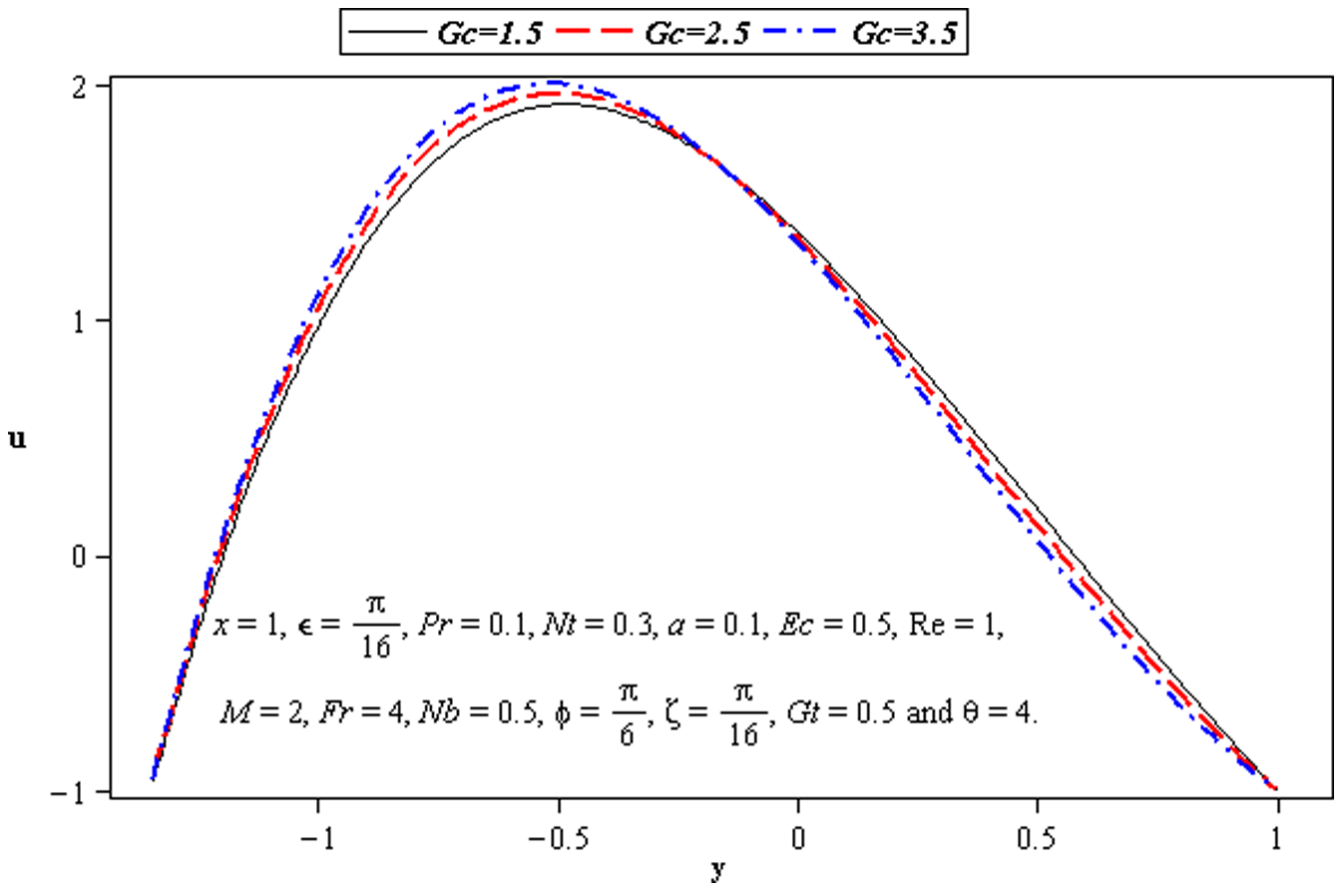
Influence of *Gc* on velocity *u*.

**Figure 6:**
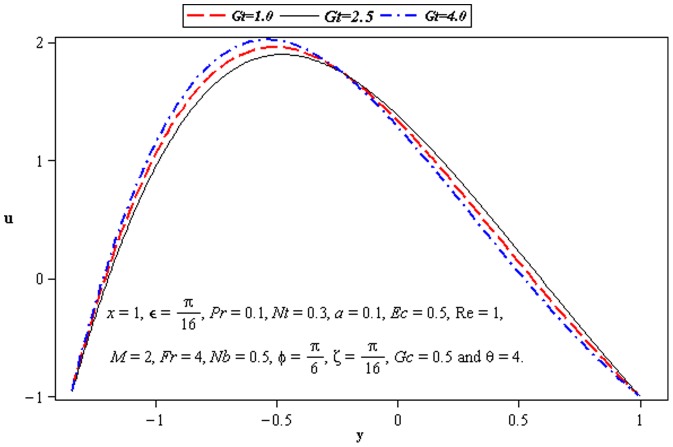
Influence of *Gt* on velocity *u*.

**Figure 7:**
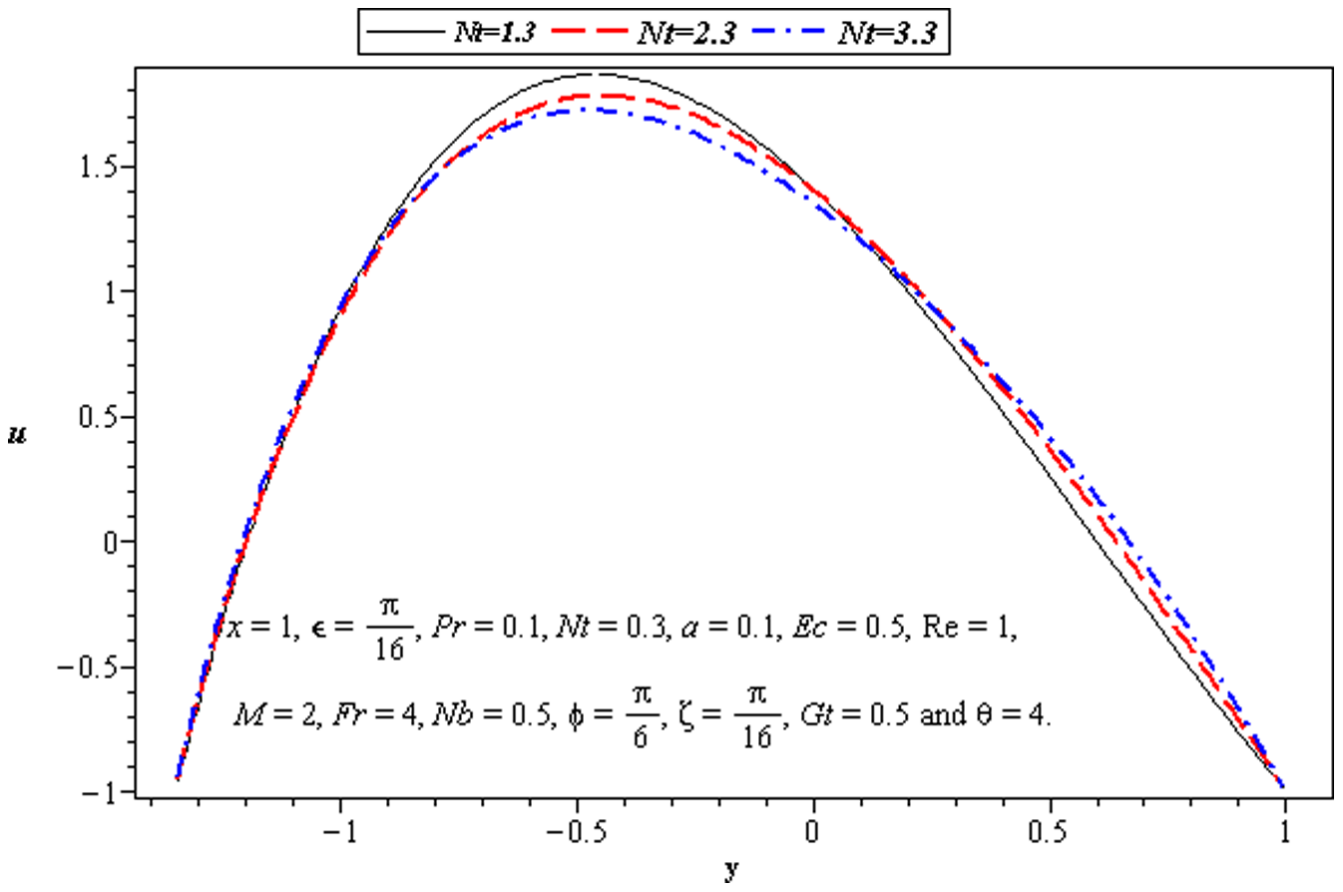
Influence of *M* on velocity *u*.

The variations of 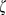, 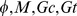, and 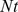 on the velocity have been seen in this subsection. Figs. 4–7 are constructed to serve the purpose. We observe that flow is more slanted towards the lower wall of channel (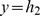) due to the consideration of inclined channel and inclined magnetic field. There is an increase in velocity at the upper wall of the channel when 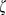 increases. Fig. 4 depicts that magnitude of the velocity of nanofluid increases at the lower wall of channel, as the values of phase difference increases (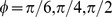). That is an increase in asymmetry leads to an increase in the fluid velocity at the lower wall of channel. Velocity *u* is decreasing function of *M* near the upper half of channel. Figs. 5 and 6 portray the power of temperature and mass Grashof number. Clearly the velocity increases near the lower wall. There is a considerable variation near the walls 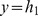 and 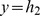 for 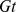 and 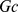. We observe that heat and mass convection supports flow near lower wall due to inclined channel. Increase in 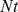 also supports the motion near the upper wall of channel which is shown in Fig. 7. This is due to thermophoretic diffusion.

### 3.3 Heat transfer characteristics

**Figure 8:**
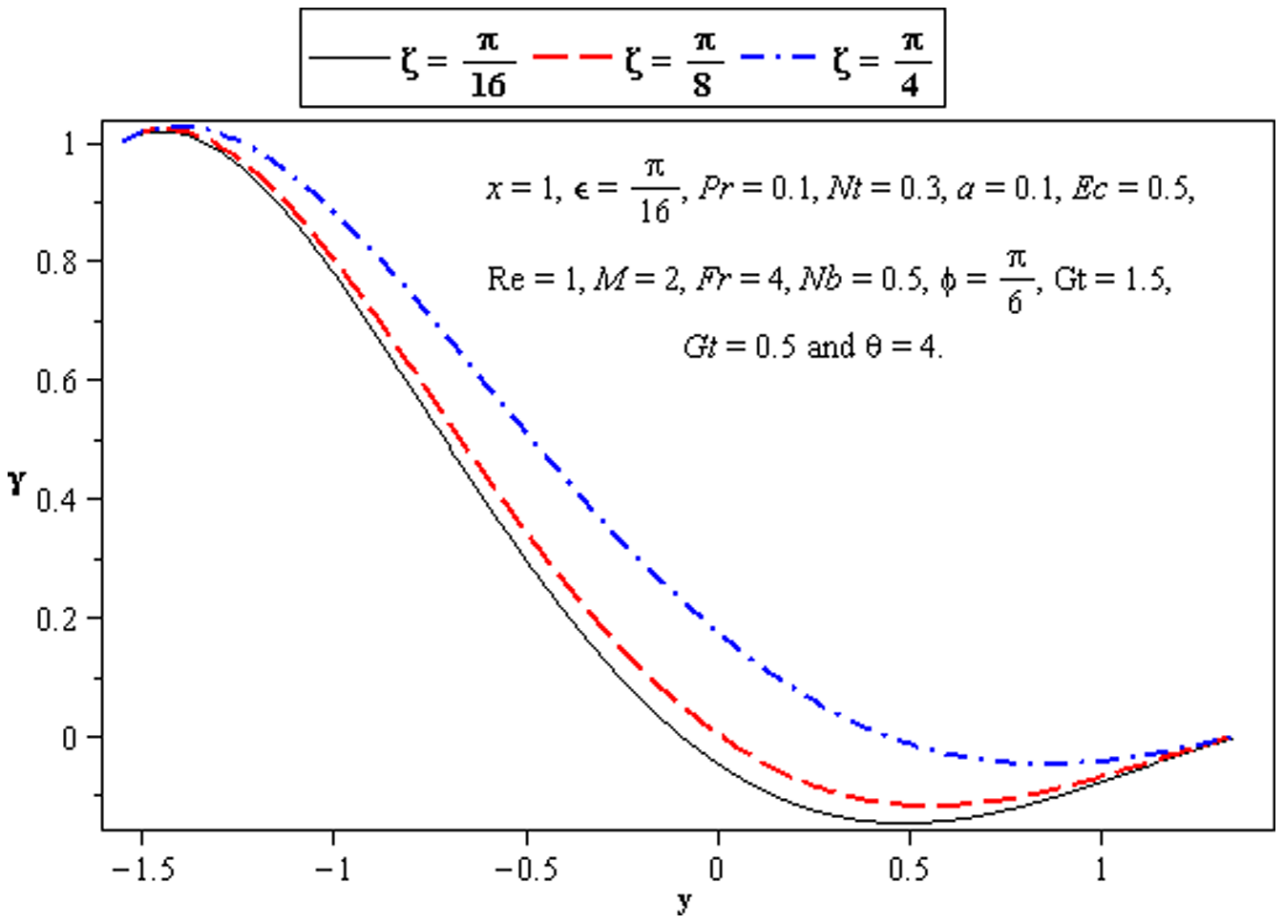
Influence of *ζ* on temperature *γ*.

**Figure 9:**
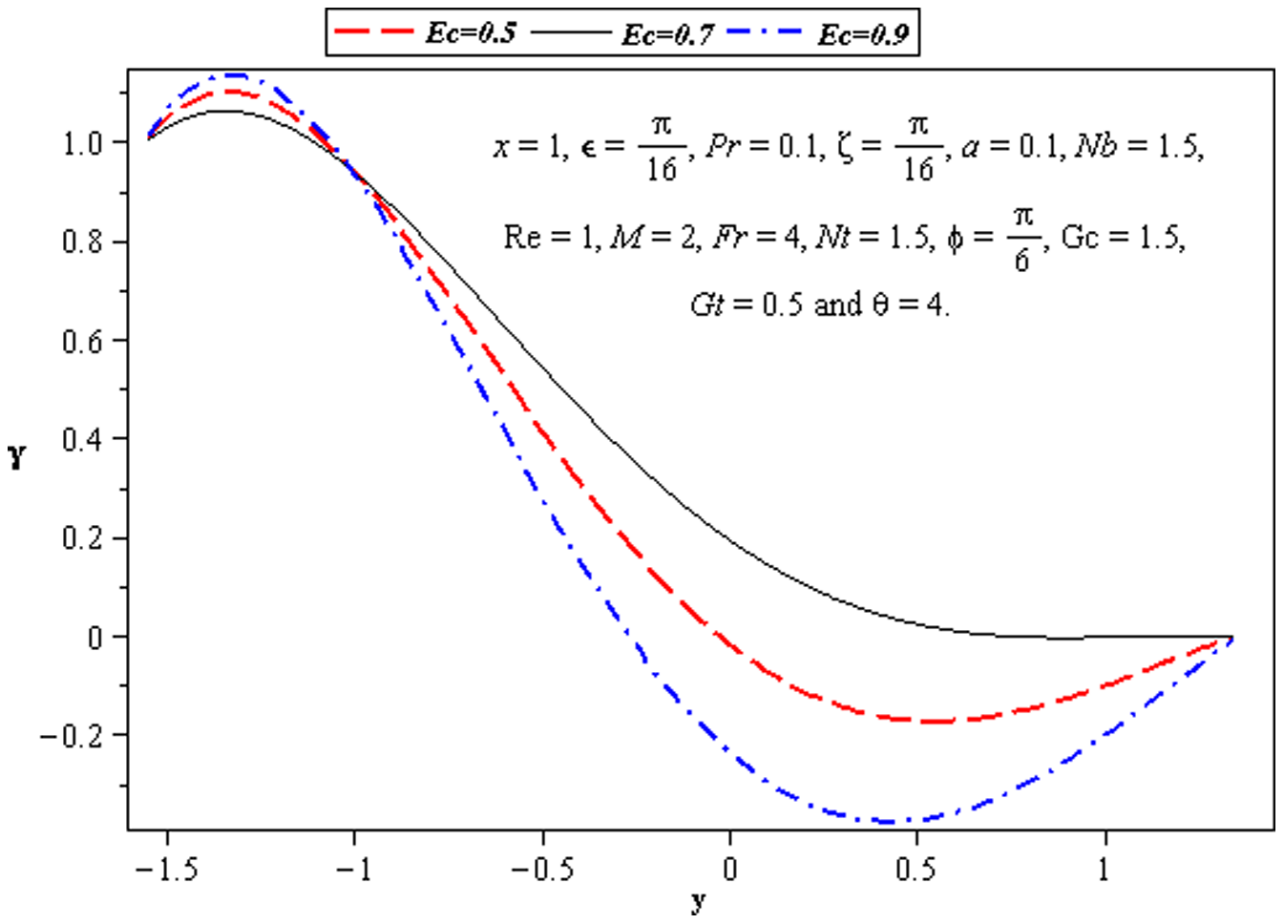
Influence of *Ec* on temperature *γ*.

**Figure 10:**
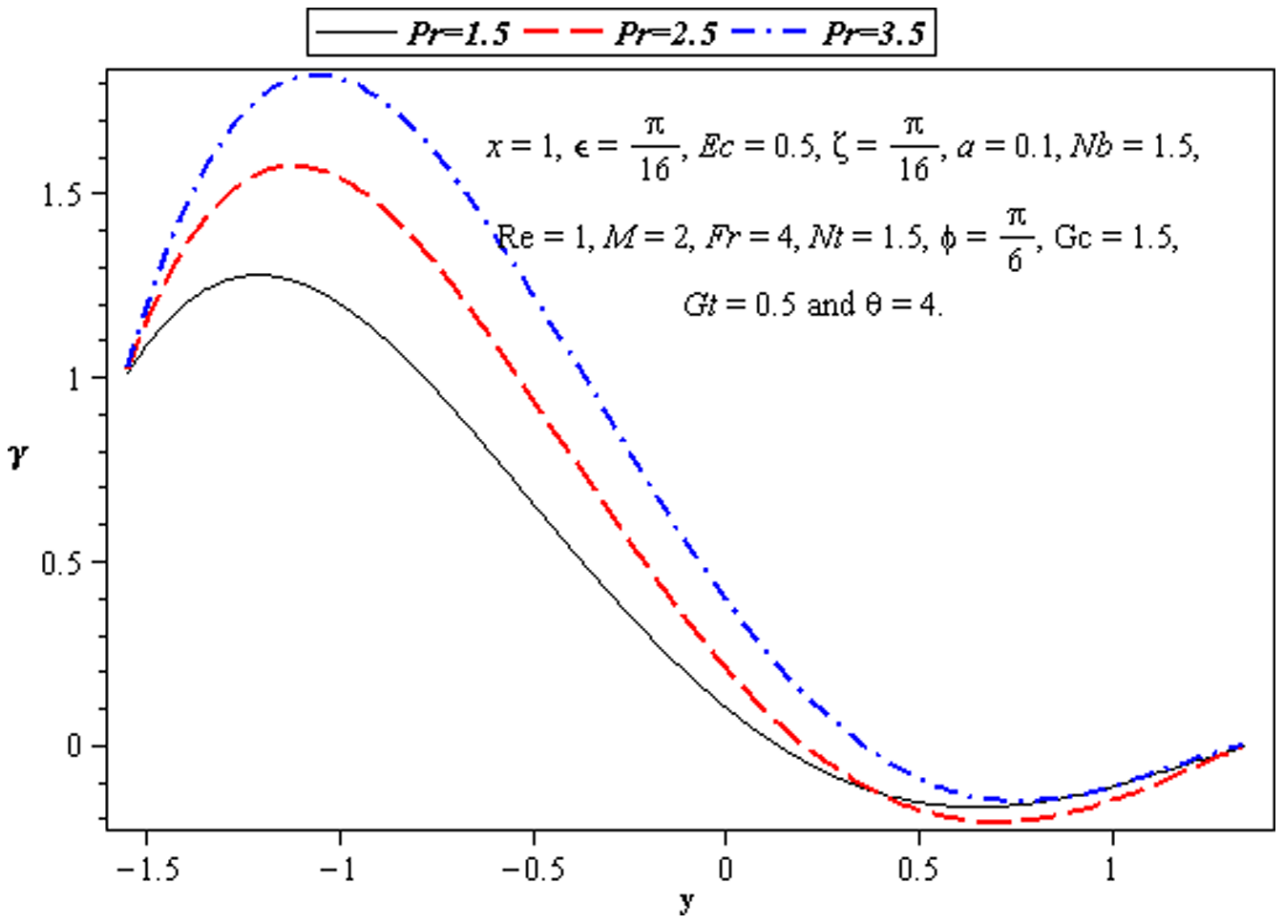
Influence of Pr on temperature *γ*.

**Figure 11:**
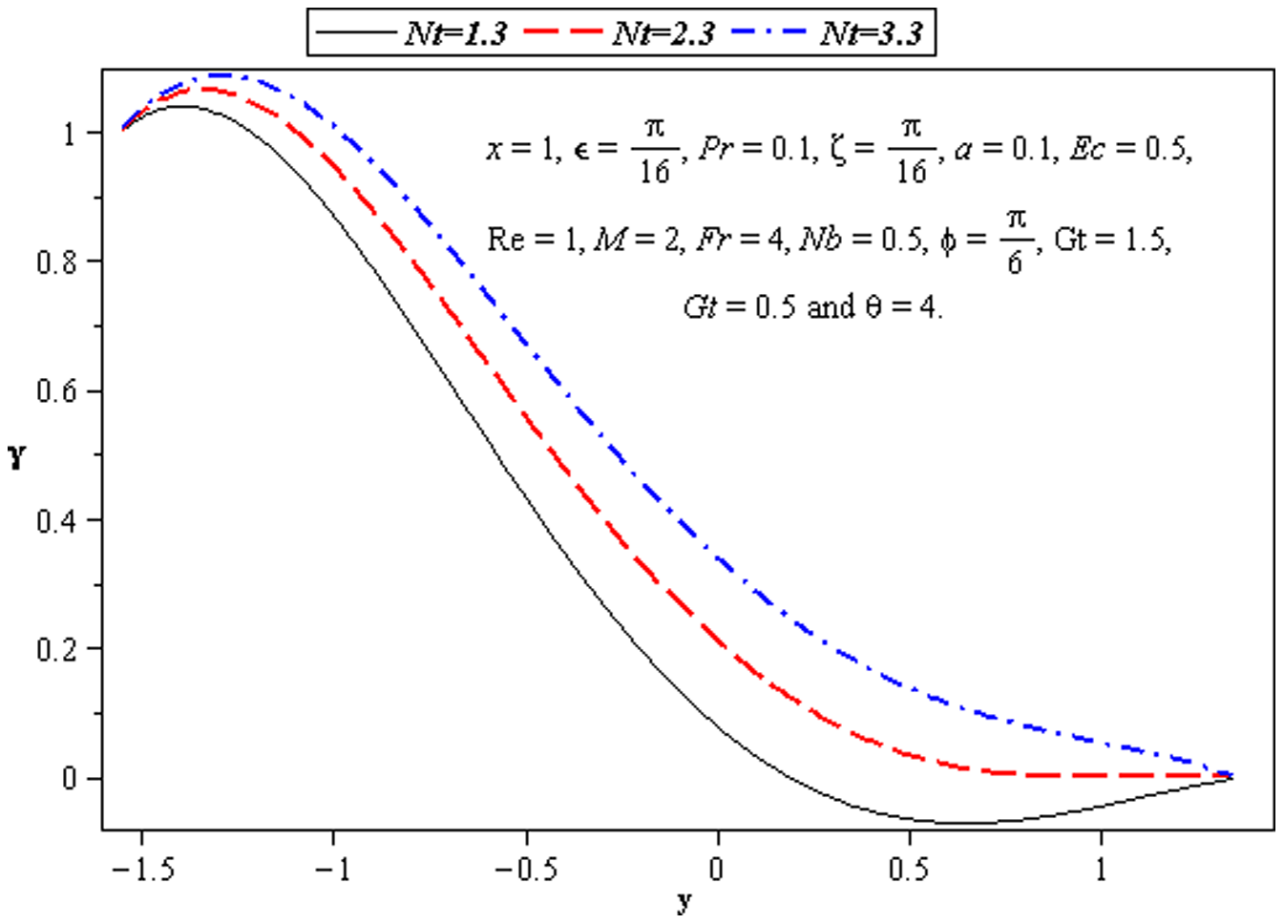
Influence of *Nt* on temperature *γ*.

**Figure 12:**
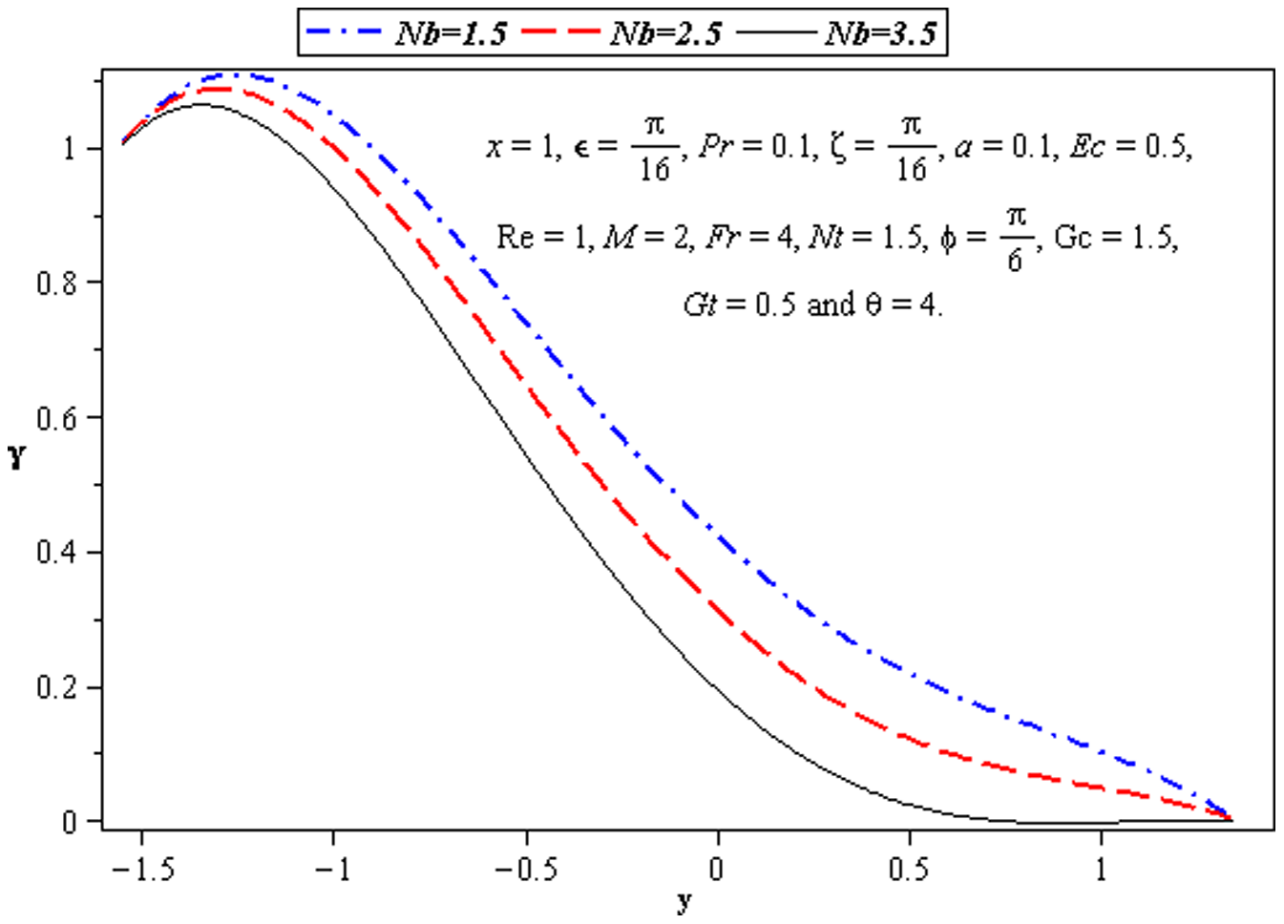
Influence of *Ec* on temperature *γ*.

Effect of heat transfer on peristalsis is shown in the Figs. 8–12. In Fig. 8, we observed the effects of 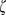 on the temperature profile 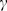 by fixing the other parameters. This Fig. indicates that the temperature increases with the increase of 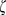. It is noticed from Figs. 9 and 10 that 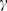 increases with 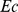 and 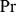 by fixing the values of other parameters. Figs. 11 and 12 depict the effects of Brownian motion parameter (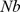) and thermophoresis parameter (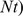 on the temperature profile. One can observe that the temperature profile is an increasing function of 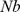 and 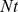 between the walls 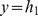 and 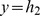. Influence of 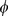 on 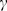 is similar to 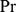 at the lower wall.

### 3.4 Mass transfer characteristics

**Figure 13:**
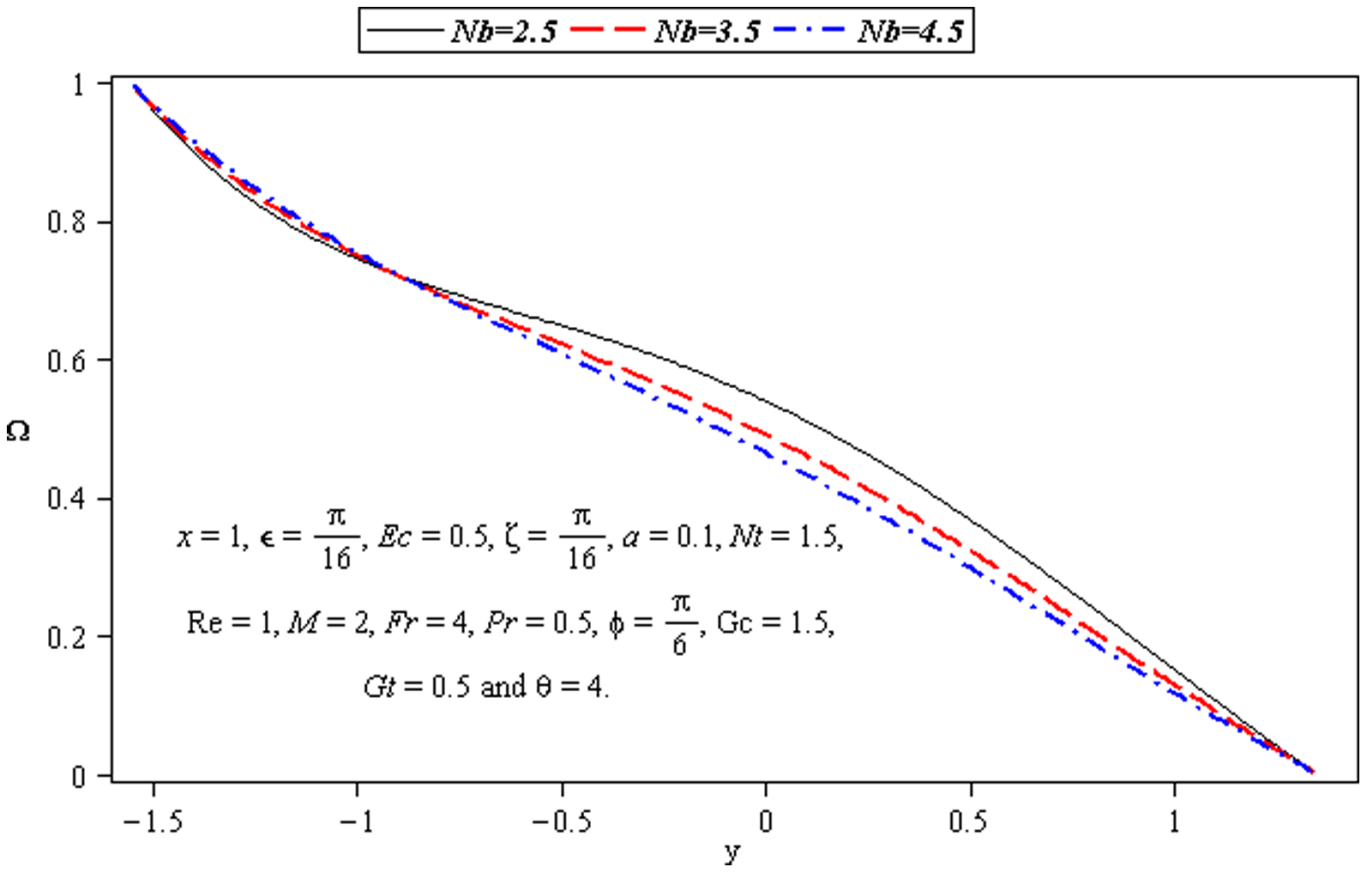
Influence of *Nb* on concentration Ω.

**Figure 14:**
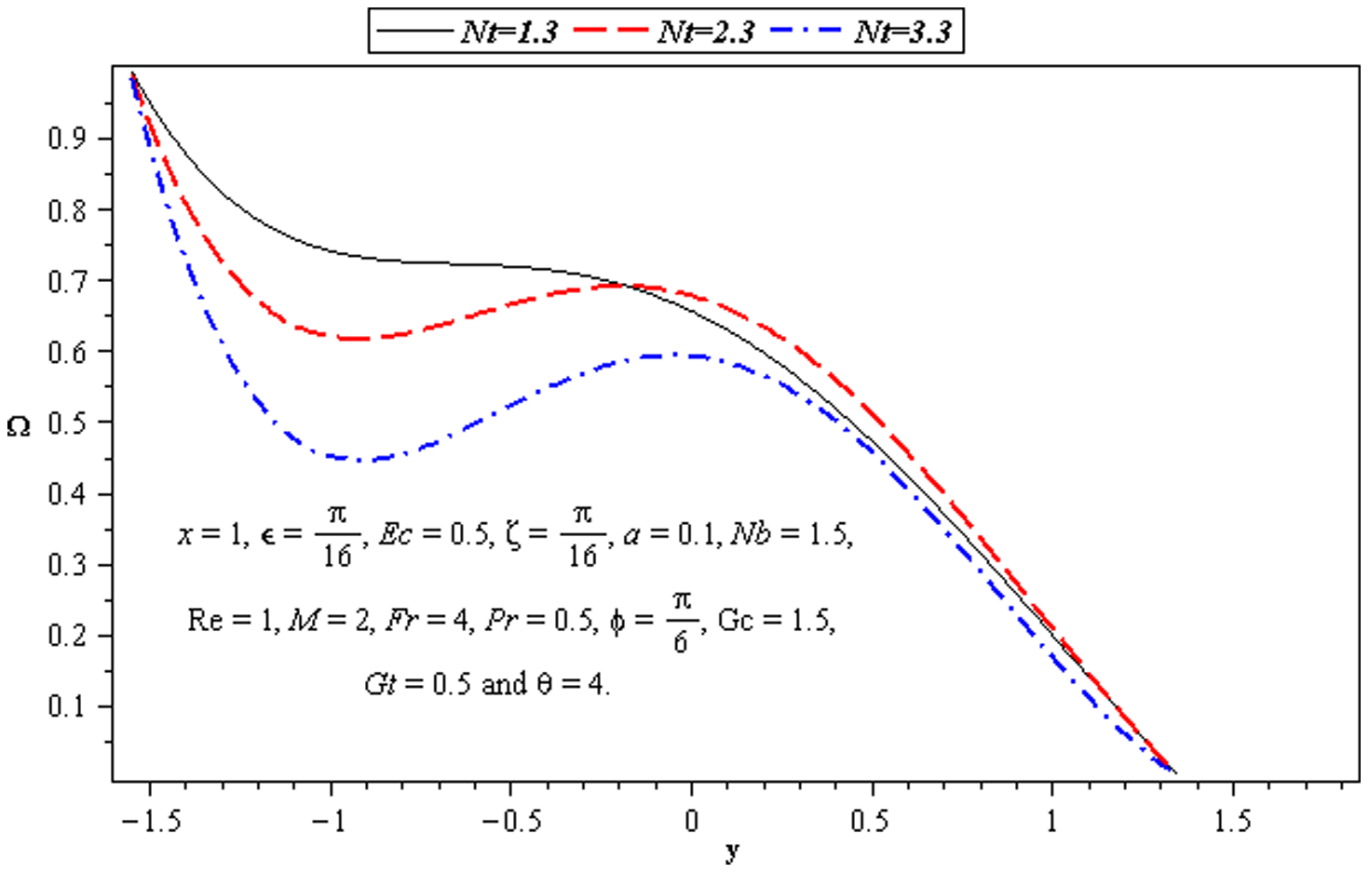
Influence of *Nt* on concentration Ω.

**Figure 15:**
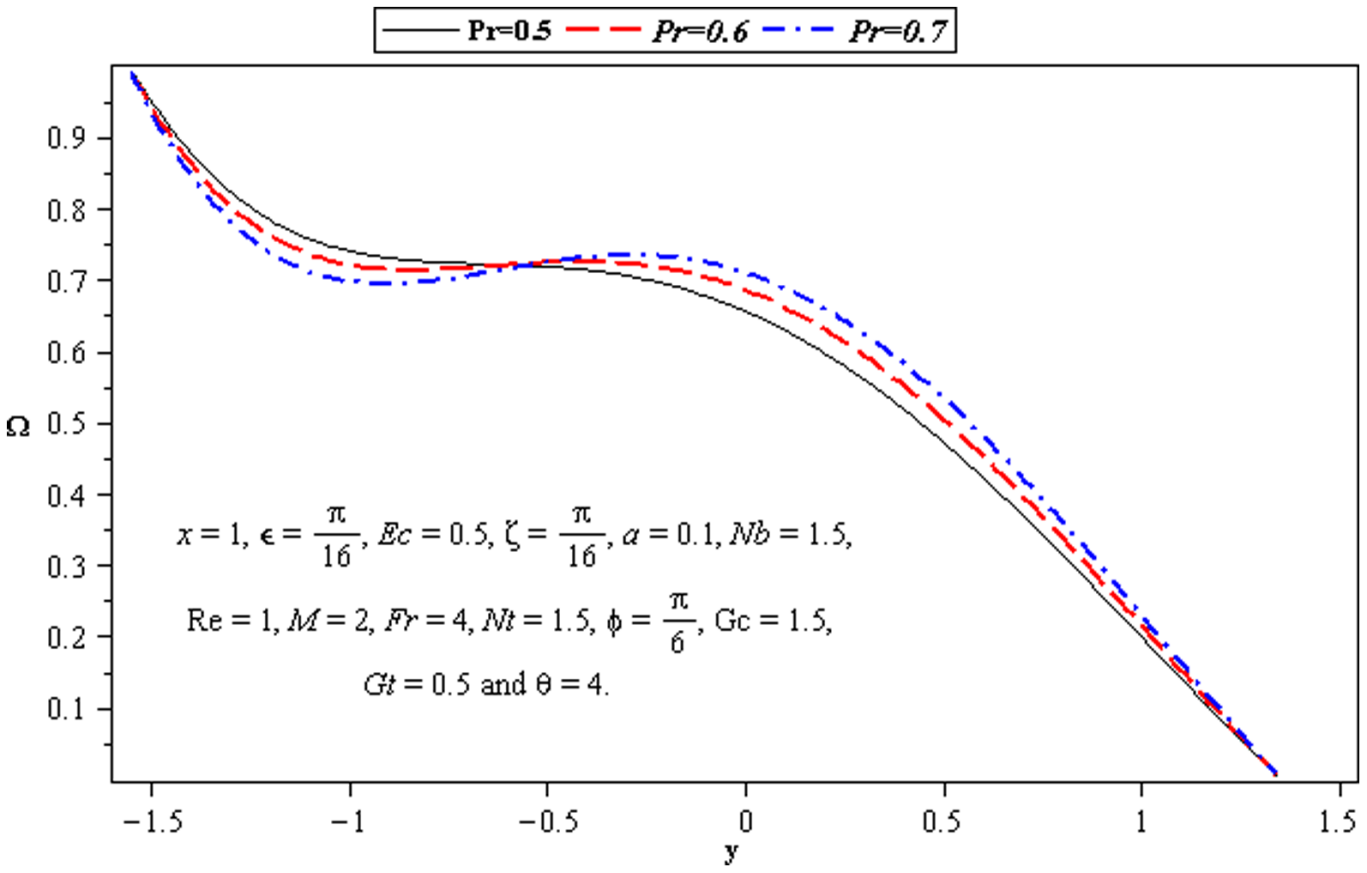
Influence of Pr on concentration Ω.

Influence of mass transfer on peristalsis is shown in the Figs. 13–15. The main parameters influencing the mass transfer include 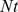 and 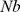. Figs. 13 and 14 depict that the concentration distribution decreases near the lower wall of channel when 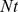 and 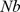 are increased. Fig. 15 illustrates that the influence of 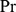 on 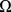 is opposite to 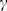 near the upper wall of channel. Since the ratio of momentum diffusivity and thermal diffusivity is inversely proportional to mass distribution.

### 3.5 Trapping

**Figure 16:**
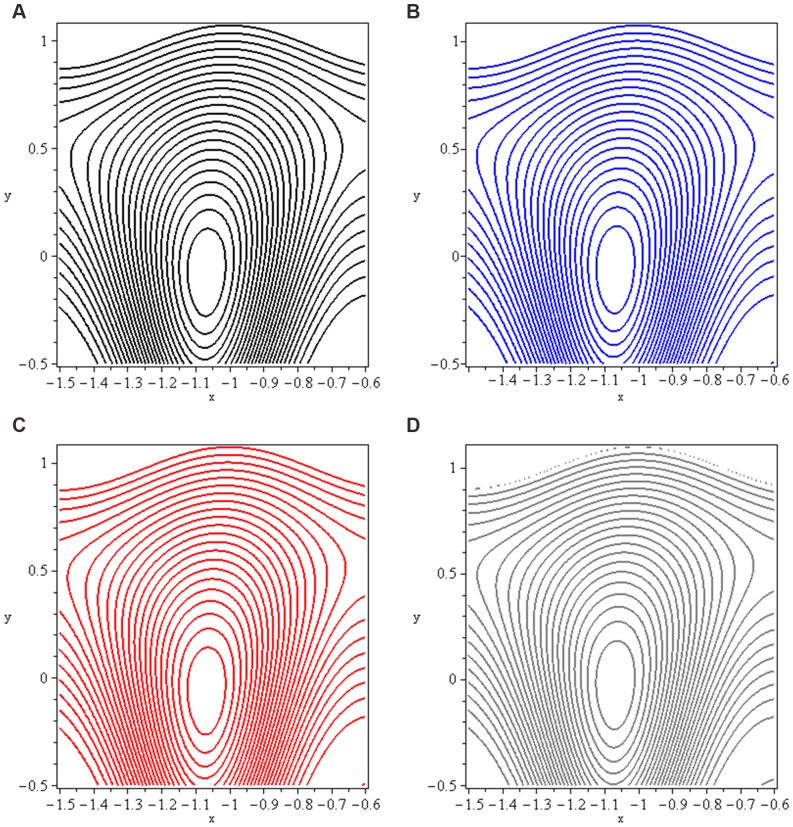
Streamlines for *Nt* (a): 0, (b): 5, (c): 10 and (d): 15.

**Figure 17:**
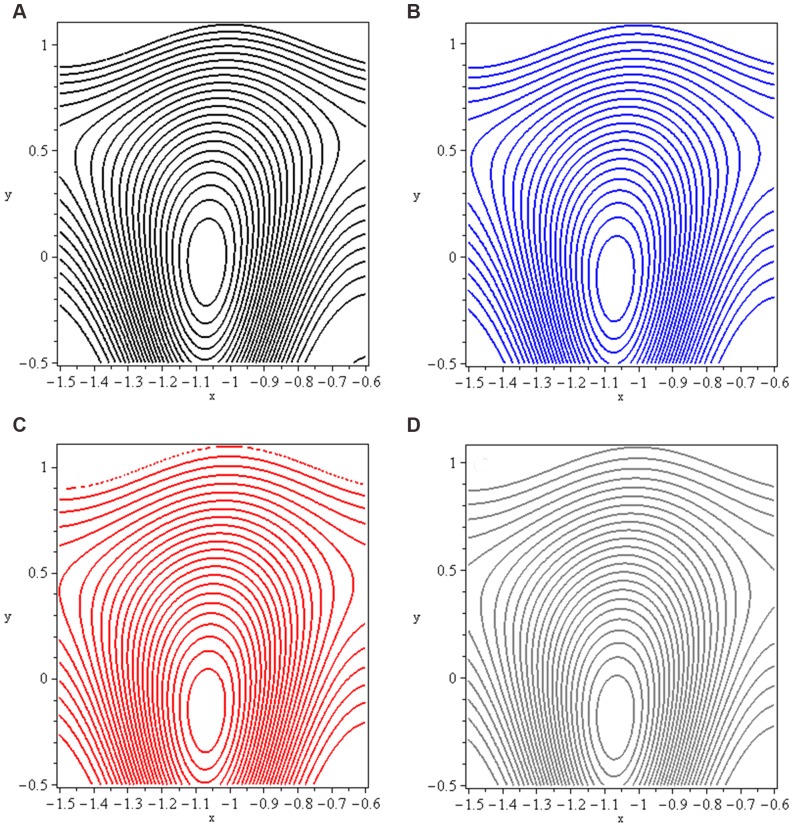
Streamlines for *Gc* (a): 0, (b): 5, (c): 10 and (d): 15.

Trapping phenomenon is shown in Figs. 16–17 for different values of 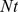 and 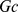 respectively. Trapping is an important aspect of peristaltic motion. It is the formation of a bolus of fluid by the closed streamlines. The case 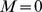 corresponds to trapping in the absence of applied magnetic field. Here we observed that bolus exists in upper part of channel. Later on, as we move towards hydromagnetic flow (increase the values of 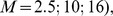 a shift towards lower half of channel is observed. Meanwhile size of trapped bolus decreases. Trapping exists for 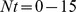 at the centre of channel. It is observed that number of closed streamlines circulating the bolus reduce in number as we increase the values of mass Grashof number.

## Conclusions

A detailed analysis is presented for magnetohydrodynamic peristaltic transport of nanofluid in an inclined asymmetric channel with heat and mass transfer. Numerical simulation is utilized for solution analysis. The critical cases from asymmetric to symmetric channel (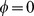), inclined to straight channel (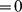), inclined hydromagnetic flow to hydromagnetic flow (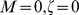) are also discussed. The main findings of the presented study are listed as follows. Pumping rate increases with 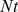 and 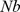 in all pumping regions. The parabolic nature of velocity distribution is disturbed due to inclined channel. Flow is more slanted towards the lower wall. Magnitude of velocity is larger in an inclined asymmetric channel than symmetric channel. Temperature distribution is an increasing function of Brownian motion parameter (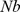) and thermophoresis parameter (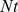).
