# Correction: Mixed Convection Flow of Nanofluid in Presence of an Inclined Magnetic Field

**DOI:** 10.1371/annotation/8c69b563-486a-4405-b71a-a2caf809ce28

**Published:** 2013-10-25

**Authors:** Saima Noreen, Bashir Ahmad, Tasawar Hayat

The correct name of the second author is: Bashir Ahmad

The correct citation is: Noreen S, Ahmad B, Hayat T (2013) Mixed Convection Flow of Nanofluid in Presence of an Inclined Magnetic Field. PLoS ONE 8(9): e73248. doi:10.1371/journal.pone.0073248

The correct version of the grant number in the first sentence of the Funding statement should be: 25-130/1433 HiCi 

